# Justifying gender discrimination in the workplace: The mediating role of motherhood myths

**DOI:** 10.1371/journal.pone.0190657

**Published:** 2018-01-09

**Authors:** Catherine Verniers, Jorge Vala

**Affiliations:** 1 Paris Descartes University, Sorbonne Paris Cité, Paris, France; 2 Institute of Social Sciences, University of Lisbon, Lisbon, Portugal; Northwestern University, UNITED STATES

## Abstract

The issue of gender equality in employment has given rise to numerous policies in advanced industrial countries, all aimed at tackling gender discrimination regarding recruitment, salary and promotion. Yet gender inequalities in the workplace persist. The purpose of this research is to document the psychosocial process involved in the persistence of gender discrimination against working women. Drawing on the literature on the justification of discrimination, we hypothesized that the myths according to which women’s work threatens children and family life mediates the relationship between sexism and opposition to a mother’s career. We tested this hypothesis using the Family and Changing Gender Roles module of the International Social Survey Programme. The dataset contained data collected in 1994 and 2012 from 51632 respondents from 18 countries. Structural equation modellings confirmed the hypothesised mediation. Overall, the findings shed light on how motherhood myths justify the gender structure in countries promoting gender equality.

## Introduction

The latest release from the World Economic Forum—the Gender Gap Report 2016 [1]–indicates that in the past 10 years, the global gender gap across education and economic opportunity and politics has closed by 4%, while the economic gap has closed by 3%. Extrapolating this trajectory, the report underlines that it will take the world another 118 years—or until 2133 –to close the economic gap entirely. Gender inequalities are especially blatant in the workplace. For instance, on average women are more likely to work part-time, be employed in low-paid jobs and not take on management positions [2, 3].

There is evidence that gender inequalities in the workplace stem, at least in part, from the discrimination directed against women. Indeed, several studies have documented personal discrimination against women by decision makers (for meta-analyses see [4, 5], some of them having more specifically examined the role of the decision makers’ level of sexist attitudes on discriminatory practices. For instance, Masser and Abrams [6] found in an experimental study that the higher the participants scored in hostile sexism, the more they were likely to recommend a male candidate rather than a female one for a managerial position. In spite of consistent evidence that higher sexism is related to greater bias toward working women [7], little is known regarding the underlying processes linking sexism to discrimination. This question remains an important one, especially because the persistence of gender discrimination contradicts the anti-discrimination rules promoted in modern societies. In fact, the issue of gender equality in employment has given rise to numerous policies and institutional measures in advanced industrial countries, all aimed at tackling gender discrimination with respect to recruitment, promotion and job assignment. In the USA, for instance, the 1964 Civil Rights Act and the 1963 Equal Pay Act provided the legal foundation for the implementation of anti-discrimination laws within the workplace. The Treaty on the European Union and the Charter of Fundamental Rights of the EU, all contain provisions relating to the promotion of equality between women and men in all areas, and the prohibition of discrimination on any ground, including sex. The member states of the European Union must comply with these provisions [8]. In this respect, some countries have incorporated legislation on equal treatment of women and men into general anti-discrimination laws (e.g., Austria, Bulgaria, Czech Republic, Germany, Ireland, Poland, Slovenia, Sweden, Great Britain), while other countries have opted for a specific gender equality act (e.g., Spain). Comparable policies have been implemented in the Asian-Pacific area, with countries including gender equality into broad anti-discrimination laws (e.g., Australia), and other countries having passed laws especially dedicated to addressing discrimination against women (e.g., Japan, the Philippines). The purpose of this research is to further explore the psychosocial process involved in the stubborn persistence of gender discrimination in the workplace, using a comparative and cross-sectional perspective of national representative samples.

### Psychosocial processes involved in justified discrimination

According to several lines of research [9–13], the expression of prejudice in contexts where social and political anti-discrimination values are prevalent implies justifications. Crandall and Eshleman [10] defined justifications as “any psychological or social process that can serve as an opportunity to express genuine prejudice without suffering external or internal sanction”. According to social dominance theory, justification of practices that sustain social inequality arises through the endorsement of legitimizing myths [13]. Moreover, research conducted in the field of system justification theory has extensively documented an increased adherence to legitimizing ideologies (including social stereotypes, meritocracy, political conservatism, etc.) in contexts where motivation to justify unequal social arrangements is heightened [14–17]. Relying on this literature Pereira, Vala and Costa-Lopes [18] provided evidence of the mediational role of myths about social groups on the prejudice-support for discriminatory measures relationship. Specifically, they demonstrated that the myths according to which immigrants take jobs away from the host society members and increase crime rates mediated the relationship between prejudice and opposition to immigration (see also [19]). We assume that an equivalent mediational process underlies the justification of gender discrimination in the workplace or, put differently, that the sexism-opposition to women’s career relationship is mediated by legitimizing myths. Glick and Fiske [20] conceptualised sexism as a multidimensional construct that encompasses hostile and benevolent sexism, both of which having three components: paternalism, gender differentiation and heterosexuality. We suspect that the gender differentiation component of sexism in particular may be related to gender discrimination in the workplace, because the maintenance of power asymmetry through traditional gender roles is at the core of this component [20]. Accordingly, it is assumed that the higher the endorsement of sexist attitudes regarding gender roles in the family, the higher the opposition to women’s work. In support of this assumption, Glick and Fiske [21] stated that gender roles are part of the more general interdependence between women and men occurring in the context of family relationships and, importantly, that these traditional, complementary gender roles shape sex discrimination. However, given that the expression of hostility towards women became socially disapproved [22, 23] and that gender discrimination in the workplace is subjected to sanctions (see for instance [24]), the release of sexism with regard to women’s role in the family and women’s professional opportunities may require justification [10, 19].

### Motherhood myths as a justification for gender discrimination

Compared with other intergroup relations, gender relations present some unique features (e.g., heterosexual interdependence; [25,26] and accordingly comprise specific myths and ideologies aimed at maintaining the traditional system of gender relations [27–29]. For instance, the belief that marriage is the most meaningful and fulfilling adult relationship appears as a justifying myth, on which men and women rely when the traditional system of gender relations is challenged by enhanced gender equality measured at the national level [30]. Drawing on this literature, we propose that beliefs that imbue women with specific abilities for domestic and parental work ensure that the traditional distribution of gender roles is maintained. In particular, we suggest that motherhood myths serve a justification function regarding gender discrimination against women in the workplace. Motherhood myths include the assumptions that women, by their very nature, are endowed with parenting abilities, that at-home mothers are bonded to their children, providing them unrivalled nurturing surroundings [31, 32]. Conversely, motherhood myths pathologised alternative mothering models, depicting employed mothers as neglecting their duty of caring, threatening the family relationships and jeopardizing mother-children bondings (see [33] for a critical review of these myths). Motherhood myths have the potential to create psychological barriers impairing women’s attempt to seek power in the workplace [34] and men’s involvement in child care [35–37]. We suggest that beyond their pernicious influence at the individual level of parental choices, motherhood myths might operate more broadly as justifications for gender discrimination regarding career opportunity. This question is of particular relevance given that equal treatment in the workplace appears even more elusive for women with children—the maternal wall [38] (see also [39–45]). At the same time, recognizing the pervasive justifying function of motherhood myths may help understand the psychosocial barriers faced not only by women who are mothers, but by women as a whole since "women are expected to become mothers sooner or later" (Dambrin & Lambert [46], p. 494; see also [47]). Relying on previous work documenting the mediational role of legitimizing myths on the prejudice—discrimination relationship [18, 19] we suggest that the myths according to which women pursuing a career threaten the well-being of the family mediates the relationship between sexist attitudes regarding gender roles and opposition to women’s work.

### Exploring gender and time as possible moderators of the hypothesized mediation

Besides the test of the main mediational hypothesis, the present research sought to explore time and gender as possible moderators of the assumed relationship between sexism, motherhood myths and discrimination. A review of the historical development of gender equality policies confirms that the implementation of laws and regulations aimed at eliminating gender discrimination in the workplace is a lengthy process (e.g., for the European countries see [48]; for the USA see [49]). In fact, although the basic principle of anti-discrimination has been enacted by many countries in the second half of the 20^th^ century, some measures are still adopted nowadays, such as the obligation for employers to publish information by 2018 about their bonuses for men and women as part of their gender pay gap reporting, a provision recently taken by the UK government. As egalitarian principles have gradually progressed in societies, it is likely that the expression of intergroup bias has become steadily subjected to social sanction. Thus, “as with racism, normative and legislative changes have occurred in many industrialized societies that make it less acceptable to express sexist ideas openly” (Tougas, Brown, Beaton, & Joly, [50], p. 843; see also [51]). Accordingly, gender discrimination within organizations became less intense and more ambiguous [52–54]. In line with this reasoning, the use of motherhood myths as a justification for unequal career opportunities may have increased over time. Conversely, it has been suggested that along with the increasing female participation in the labour market over the last decades, a positive attitude regarding the government-initiated women-friendly policies now coexists with an adherence to traditional family values and norms [55]. There is a possibility that the coexistence of contradictory norms in the same culture may leave some room for the expression of gender bias (i.e., a normative compromise, [56]), reducing slightly the need to rely on justifications to discriminate against working women. The present research will examine these possibilities by studying the role of motherhood myths on the sexism—discrimination relationship in 1994 and 2012.

Another possible moderator examined in the present study is the respondents' gender. Basically, the reason why people rely on justifications is to express their genuine prejudices without appearing biased. Consistent evidence, however, suggests that the perpetrator’s gender affects people’s perception of sexism towards women: given that sexism is generally conceived as involving a man discriminating against a woman, men are perceived as prototypical of the perpetrator [57, 58]. As a consequence, sexist behaviours carried out by males are perceived as more sexist than the same behaviours enacted by females [59, 60]. Moreover, the expression of sexism by women may go undetected due to the reluctance of women to recognize that they might be harmed by a member of their own gender group [22]. Taken together, these findings suggest that a woman is more likely than a man to express sexist bias without being at risk of appearing sexist. In line with this reasoning, one could assume that men need to rely on justifications to discriminate to a greater extent than women do. Alternatively, women expressing sexism against their ingroup members are at risk of being negatively evaluated for violating the prescription of feminine niceness [61, 62]. As a consequence, women might be inclined to use justifications to discriminate in order to maintain positive interpersonal evaluations. An additional argument for assuming that women may rely on motherhood myths lies in the system justification motive. According to system justification theory [63, 64], people are motivated to defend and justify the status quo, even at the expense of their ingroup. From this perspective, the belief that every group in society possesses some advantages and disadvantages increases the belief that the system is balanced and fair [29, 65]. Motherhood myths imbue women with a natural, instinctual and biologically rooted capacity to raise children that men are lacking [66]. In addition, they convey gender stereotype describing women in positive terms (e.g., considerate, warm, nurturing) allowing a women-are-wonderful perception [27]. As a consequence, women are likely to rely on motherhood myths to restore the illusion that, despite men structural advantage [67, 68], women as a group still possess some prerogatives [34].

### Overview

The aim of the present study is to test the main hypothesis (H1) that motherhood myths are a justification that mediates the relationship between sexism and opposition to women’s work following the birth of a child. Additionally, two potential moderators of this mediational process are considered. The present research tests the exploratory hypotheses that (H2) the assumed mediational process is moderated by time and (H3) by participants’ gender. We tested these hypotheses using the Family and Changing Gender Roles module of the International Social Survey Programme [69, 70]. This international academic project, based on a representative probabilistic national sample, deals with gender related issues, including attitudes towards women’s employment and household management. Hence this database enables a test of the proposed mediational model on a large sample of female and male respondents and data gathered 18 years apart.

## Method

### Data

We used the 2012 and 1994 waves of the ISSP Family and Changing Gender Roles cross-national survey [69, 70]. The ISSP published fully anonymized data so that individual survey participants cannot be identified. The two databases slightly differed regarding the involved countries, some of which did not participate in the two survey waves. In order to maintain consistency across the analyses, we selected 18 countries that participated in both survey waves (i.e., Austria, Australia, Bulgaria, Canada, Czech Republic, Germany, Great Britain, Ireland, Israel, Japan, Norway, Philippines, Poland, Russia, Slovenia, Spain, Sweden and the USA). The data file for the 2012 survey wave included 24222 participants (54.4% female participants), mean age = 49.38, *SD* = 17.54, and the data file for the 1994 survey wave included 27410 participants (54.4% female participants), mean age = 44.26, *SD* = 17.07.

### Measures

The main variables used in this study are the following:

#### Sexism

One indicator was used to capture sexism: “A man's job is to earn money; a woman's job is to look after the home and family”. This item taps into the gender differentiation component of sexism [20, 25]. Participants answered on a 5 point likert scale ranging from 1 = strongly agree to 5 = strongly disagree. Data was recoded so that the higher scores reflected higher sexism.

#### Motherhood myths

Two indicators were used that capture the myths about the aversive consequence of mother’s work for her child and the family: “A preschool child is likely to suffer if his or her mother works” and “All in all, family life suffers when the woman has a full-time job”. Participants answered on a 5 point likert scale ranging from 1 = strongly agree to 5 = strongly disagree. Data was recoded so that the higher scores reflected higher endorsement of motherhood myths.

#### Opposition to women’s career

Two indicators were used to capture the opposition to women’s professional career following the birth of a child: “Do you think that women should work outside the home full-time, part-time or not at all when there is a child under school age?” and “Do you think that women should work outside the home full-time, part-time or not at all after the youngest child starts school?” Participants answered on a scale ranging from 1 = work full time, 2 = work part-time, 3 = stay at home.

In addition, the first step of our analyses involved the following control variables: participant’s gender and age, partnership status, educational level, subjective social status, attendance of religious services and political orientation.

## Results

The following section presents the results of a four-step analysis: The first step consists of a preliminary hierarchical regression analysis to establish the respective contributions of demographical variables, sexism and motherhood myths to opposition to women’s work. The second step is dedicated to a test of the construct validity of the proposed measurement model using Confirmatory Factor Analyses. The third step involves a test of the hypothesized mediation. Finally, the last step is a test of the hypothesized moderated mediations.

### Step 1: Hierarchical regression analysis

Inspection of the correlation matrix (Table 1) indicates that all the correlations are positive, ranging from moderate to strong. The pair of items measuring motherhood myths presents the strongest correlation (*r* (48961) = .633), followed by the pair of items measuring opposition to women’s career (*r* (45178) = .542).

**Table 1:** Means, standard deviations and correlation matrix of the indicators.

			Correlation matrix			
Indicators	*M*	*SD*	(1)	(2)	(3)	(4)
Sexism						
(1) *Man’s job earn money*, *woman’s job family*	2.75	1.30				
Motherhood myths						
(2) *Mother works child suffers*	3.10	1.22	.428			
(3) *Woman works family suffers*	3.05	1.25	.443	.633		
Opposition						
(4) *Should women work child under school age*	2.31	0.68	.337	.377	.345	
(5) *Should women work child starts school*	1.81	0.66	.384	.332	.365	.542

All coefficients are significant at *p* < .001.

We conducted a hierarchical regression analysis to establish the respective contributions of demographical variables, sexism and motherhood myths to opposition to women’s work. In block 1, participant’s gender (male = -1, female = 1) and partnership (no partner = -1, partner = 1) were entered together with standardized scores of age, years of schooling, subjective social status, attendance of religious services and political orientation. Block 2 included sexism, the myths about the aversive consequence of mother’s work for her child and for family (all standardized). Predictors in block 1 accounted for 9% of the variance, *F*(7, 10140) = 157.89, *p* < .001. The analysis revealed the significant effects of participant’s gender (*B* = -.033, *SE* = .006, *p* < .001), age (*B* = .058, *SE* = .006, *p* < .001), years of schooling (*B* = -.135, *SE* = .007, *p* < .001), subjective social status (*B* = -.057, *SE* = .007, *p* < .001), religiosity (*B* = .076, *SE* = .006, *p* < .001) and political orientation (*B* = .04, *SE* = .006, *p* < .001). Partnership was unrelated to opposition to women’s career (*B* = .002, *SE* = .006, *p* = .77). Taken together the results indicate that the higher the time of education and the subjective social status, the lower the opposition to women’s work. Conversely, the higher the age, religiosity and political conservatism, the higher the opposition to women’s work. Finally, results indicate that opposition to women’s work is more pronounced amongst men than amongst women. When entered in block 2, sexism and motherhood myths accounted for an additional 18% of the variance, indicating that these variables significantly improved the model’s ability to predict opposition to women’s work, over and above the contributions of gender, partnership, education, social status, religiosity and political orientation (Δ*R*^2^ = .18), Δ*F*(3, 10137) = 854.04, *p* < .001. Specifically, the analysis revealed the significant effects of sexism (*B* = .151, *SE* = .006, *p* < .001), myth about the aversive consequence of mother’s work for her child (*B* = .10, *SE* = .007, *p* < .001) and myth about the aversive consequence of women’s work for family (*B* = .09, *SE* = .007, *p* < .001). It should be noted that the effect of participant’s gender virtually disappeared after controlling for sexism and motherhood myths (Table 2). In addition, we performed this hierarchical regression analysis separately for the two waves and consistently found that the variables of our model (sexism and the motherhood myths) explained more variance than the demographical variables.

**Table 2:** Summary of hierarchical regression analysis for variables predicting opposition to women’s career.

Predictor	*B*	*SE B*	*B*	*R*^*2*^
Block 1				.098
Gender	-.033	.006	-.056	
Age	.058	.006	.095	
Partnership	.002^a^	.006	.003	
Education	-.135	.007	-.193	
Social status	-.057	.007	-.085	
Religiosity	.076	.006	.122	
Political orientation	.04	.006	.068	
Block 2				.280
Gender	-.003^a^	.005	-.006	
Age	.016^b^	.005	.025	
Partnership	-.001^a^	.006	-.001	
Education	-.072	.007	-.103	
Social status	-.020	.006	-.030	
Religiosity	.022	.006	.035	
Political orientation	.020	.005	.035	
Sexism	.151	.006	.244	
Motherhood myth—Child	.100	.007	.170	
Motherhood myth—Family	.090	.007	.151	

Gender is coded -1 for men and 1 for women. Partnership is coded -1 for no partner and 1 for partner. All coefficients and *Rs*^2^ are significant at the level *p* < .001, except the coefficients with superscripts:

^a^ *p* > .10,

^b^ *p* < .005.

### Step 2: Confirmatory factor analyses

We conducted a CFA to check the construct validity of the proposed measurement model. CFA and subsequent analyses were all performed using R. 3.4.1 and the Lavaan package [71]. The loading of the single indicator of the sexism variable and the loading of the first indicator of the motherhood myths and opposition variables were constrained to 1.00 [72], and the three variables were allowed to correlate. Results show a good fit to the data, χ^2^(3, *N* = 42997) = 400.36, *p* < .001, CFI = .993, RMSEA = .05 [90% CI = .05, .06], SRMR = .01, AIC = 540804. In addition, we estimated an alternative model in which all items loaded on a unique latent variable. This alternative model shows a poorer fit to the data, χ^2^(5, *N* = 42997) = 8080.28, *p* < .001, CFI = .867, RMSEA = .19 [90% CI = .19, .19], SRMR = .07, AIC = 548480. The comparison of the two models indicates that the proposed measurement model fits the data better than the alternative one, Δ χ^2^ (2, 42997) = 7679.9, *p* < .001. We repeated this comparison in each country and results confirm that the proposed measurement model fits better in all countries (see S1 Table for comparative test of the goodness of fit of the hypothesized measurement model vs. alternative measurement model in each country).

We tested the measurement invariance of the CFA model across the two survey waves. To do this, we conducted a model comparison to test for configural and metric invariances. Results indicate that the configural invariance can be retained, χ^2^(6, *N* = 42997) = 513.05, *p* < .001, CFI = .991, RMSEA = .06 [90% CI = .05, .06], SRMR = .01, AIC = 537580. When constraining the loadings to be equal across waves fit indices remain satisfactory, χ^2^(8, *N* = 42997) = 679.58, *p* < .001, CFI = .989, RMSEA = .06 [90% CI = .05, .06], SRMR = .02, AIC = 537743. The change in CFI is below the cutpoint of .01, indicating that the metric invariance can be retained and that further comparisons of the relationships between constructs across survey waves can be performed [73, 74]. Furthermore, we repeated this comparison in each country and results support the configural invariance of the CFA model across survey waves in all countries. In addition, the full metric invariance is obtained in all but three countries—Poland, Slovenia and the USA. In these countries, the CFIs are larger than the cutpoint of .01, indicating a lack of full metric invariance. Nonetheless, we were able to retain a partial metric invariance of the CFA model across the survey waves by setting free one non-invariant loading [75], (see S2 Table for the test of the invariance of the measurement model across survey waves by country).

We tested the measurement invariance of the CFA model across gender groups using the same procedure as for the test of the measurement invariance across survey waves. The baseline model constraining the factor structure to be equal in the two gender groups shows good fit to the data, χ^2^(6, *N* = 42943) = 440.95, *p* < .001, CFI = 0.993, RMSEA = .05 [90% CI = .05, .06], SRMR = .01, AIC = 539573, indicating that the configural invariance is achieved for the two groups. Then we fitted a more restricted model in which the factor loadings were constrained to be equal across groups. This model allows testing for the metric invariance (equal loadings) of the model across gender. Once again, the results indicate that this constrained model show good fit to the data, χ^2^(8, *N* = 42943) = 469.14, *p* < .001, CFI = 0.992, RMSEA = .05 [90% CI = .04, .05], SRMR = .01, AIC = 539598. Furthermore, the Δ CFI is below the cutpoint of .01, indicating that the metric invariance can be retained [75]. This result confirms that cross gender comparisons of the relationships between constructs can reasonably be performed. Furthermore, we repeated this procedure in each country. Once again, the Δ CFIs are below the cutpoint of .01, indicating that the configural invariance of the CFA model across gender groups is achieved in all countries (see S3 Table for the test of the invariance of the measurement model across gender groups by country).

### Step 3. Mediation analysis

#### Overview of the analysis strategy

This study main hypothesis is that (H1) the more people hold sexist attitude regarding gender roles, the more they endorse motherhood myths, which in turn enhances the opposition to women’s career after the birth of a child. In order to test this assumption, we ran mediational analyses using structural equation modelling. First, we examined the goodness of fit of the hypothesized mediational model and compared it with the goodness of fit of two alternative models. In the first alternative model, motherhood myths predict sexism that, in turn, predicts opposition. In the second alternative model, opposition to women’s career predicts motherhood myths. After having established that the hypothesized model adequately fit the data, we examined the coefficients for the hypothesized relationships between variables.

#### Goodness of fit of the models

Inspection of the fit indices indicates that the hypothesized model fits the data better than the first alternative model in 16 out of the 18 analysed countries (Table 3). Thus, in these countries the data is better accounted for by a model stating motherhood myths as a mediator of the sexism-opposition to women’s career relationship, rather than by a model stating sexism as a mediator of the myths-opposition to women’s career relationship. The comparison of the fit indices indicates that the two models fit the data to almost the same extent in the two remaining countries (i.e., Czech Republic, and Philippines). Finally, the second alternative model—where opposition to women’s career predicted motherhood myths and sexism—shows very poor fit to the data in all countries. This result suggests that endorsement of motherhood myths is not a mere consequence of discrimination.

**Table 3:** Goodness-of-fit indices for the hypothesized mediational model and alternative models by country.

Country	χ 2	CFI	RMSEA	SRMR	AIC	Δ χ 2
Austria						
Hypothesized model (df = 4)	91.2	.959	.10 [.09, .12]	.05	23013	
Alternative model 1 (df = 4)	350.3	.838	.21 [.19, .23]	.13	23273	
Alternative model 2 (df = 5)	658.16	.694	.26 [.24, .28]	.20	23578	566.9
Australia						
Hypothesized model (df = 4)	110.58	.978	.09 [.08, .11]	.05	30161	
Alternative model 1 (df = 4)	634.5	.868	.24 [.22, .25]	.14	30685	
Alternative model 2 (df = 5)	1024.1	.787	.27 [.25, .28]	.22	31072	913.4
Bulgaria						
Hypothesized model (df = 4)	18.82	.989	.04 [.02, .06]	.01	24157	
Alternative model 1 (df = 4)	199.7	.851	.16 [.14, .18]	.10	24337	
Alternative model 2 (df = 5)	290.97	.782	.18 [.16, .19]	.13	24427	272.1
Canada						
Hypothesized model (df = 4)	56.82	.985	.08 [.06, .10]	.04	21367	
Alternative model 1 (df = 4)	312.84	.911	.20 [.18, .22]	.11	21623	
Alternative model 2 (df = 5)	736.87	.789	.28 [.26, .30]	.23	22045	680.0
Czech Republic						
Hypothesized model (df = 4)	17.14	.995	.03 [.02, .05]	.01	32741	
Alternative model 1 (df = 4)	124.8	.958	.10 [.09, .12]	.06	32849	
Alternative model 2 (df = 5)	370.17	.874	.17 [.15, .18]	.13	33092	353.04
Germany						
Hypothesized model (df = 4)	238.7	.971	.11 [.10, .13]	.06	51502	
Alternative model 1 (df = 4)	1123.6	.861	.25 [.24, .26]	.15	52387	
Alternative model 2 (df = 5)	1771.5	.781	.28 [.27, .29]	.23	53033	1532.8
Great Britain						
Hypothesized model (df = 4)	77.57	.971	.10 [.08, .13]	.04	16910	
Alternative model 1 (df = 4)	304.26	.881	.22 [.20, .24]	.12	17137	
Alternative model 2 (df = 5)	616.97	.757	.28 [.26, .30]	.22	17447	539.4
Ireland						
Hypothesized model (df = 4)	61.39	.982	.09 [.07, .11]	.05	20300	
Alternative model 1 (df = 4)	315.71	.900	.21 [.19, .23]	.13	20554	
Alternative model 2 (df = 5)	712.91	.772	.28 [.27, .30]	.23	20949	651.52
Israel						
Hypothesized model (df = 4)	21.06	.993	.04 [.02, .06]	.01	26053	
Alternative model 1 (df = 4)	237.67	.901	.16 [.14, .18]	.10	26269	
Alternative model 2 (df = 5)	505.04	.788	.21 [.19, .22]	.16	26592	483.98
Japan						
Hypothesized model (df = 4)	29.12	.983	.05 [.03, .07]	.02	26340	
Alternative model 1 (df = 4)	115.31	.925	.12 [.10, .14]	.07	26426	
Alternative model 2 (df = 5)	214.43	.859	.14 [.13, .16]	.10	26523	185.31
Norway						
Hypothesized model (df = 4)	77.97	.989	.07 [.06, .09]	.03	32441	
Alternative model 1 (df = 4)	718.14	.892	.24 [.23, .26]	.13	33081	
Alternative model 2 (df = 5)	1558.4	.764	.32 [.31, .33]	.27	33920	1480.5
Philippines						
Hypothesized model (df = 4)	19.01	.984	.04 [.02, .06]	.01	29706	
Alternative model 1 (df = 4)	40.49	.961	.06 [.04, .08]	.03	29728	
Alternative model 2 (df = 5)	180.81	.814	.12 [.10, .14]	.08	29866	161.8
Poland						
Hypothesized model (df = 4)	117.24	.964	.11 [.09, .13]	.06	28495	
Alternative model 1 (df = 4)	409.59	.870	.21 [.19, .23]	.12	28788	
Alternative model 2 (df = 5)	993.28	.683	.29 [.28, .31]	.22	29369	876.04
Russia						
Hypothesized model (df = 4)	12.71	.997	.02 [.01, .04]	.01	35328	
Alternative model 1 (df = 4)	199.42	.928	.12 [.11, .14]	.07	35514	
Alternative model 2 (df = 5)	387.38	.859	.16 [.14, .17]	.12	35701	374.67
Slovenia						
Hypothesized model (df = 4)	7.25	.999	.02 [.00, .04]	.01	22547	
Alternative model 1 (df = 4)	281.77	.914	.19 [.17, .21]	.12	22821	
Alternative model 2 (df = 5)	595.08	.817	.25 [.23, .26]	.21	23133	587.83
Spain						
Hypothesized model (df = 4)	51.3	.991	.05 [.04, .06]	.01	48463	
Alternative model 1 (df = 4)	382.6	.930	.14 [.13, .16]	.09	48794	
Alternative model 2 (df = 5)	1388.5	.746	.25 [.24, .26]	.19	49798	1337.2
Sweden						
Hypothesized model (df = 4)	81.63	.979	.10 [.08, .12]	.04	20684	
Alternative model 1 (df = 4)	543.2	.856	.26 [.24, .28]	.14	21145	
Alternative model 2 (df = 5)	994.45	.735	.32 [.30, .33]	.25	21595	912.82
USA						
Hypothesized model (df = 4)	2.76	1.00	.00 [.00, .02]	.01	25311	
Alternative model 1 (df = 4)	408.53	.872	.21 [.20, .23]	.13	25717	
Alternative model 2 (df = 5)	683.44	.785	.25 [.23, .26]	.20	25990	680.68

Δ χ^2^ compares the second alternative model with the hypothesized mediational model. All Δ χ^2^ tests are significant at *p* < .001. The hypothesized mediational model and the first alternative model are not nested and therefore a Δ χ^2^ test cannot be computed.

#### Test of the relationships between variables

The goodness of fit of the proposed mediational model having been established in 16 countries out of 18, we next examined the coefficients for the hypothesized relationships in these countries. Table 4 shows the results of the mediation analysis in the 16 retained countries. The total effect of sexism on opposition to women’s career is positive and significant in all countries. The direct effect is reduced in all countries when controlling for the indirect effect through motherhood myths. As recommended in the literature, the indirect effects were subjected to follow-up bootstrap analyses using 1000 bootstrapping resamples [76]. The null hypothesis is rejected and the indirect effect is considered significant if the 95% confidence intervals (CI) do not include zero. All bias corrected 95% CI for the indirect effect excluded zero, indicating that in line with H1, endorsement of motherhood myths is a significant mediator of the relationship between sexism and opposition to women’s career in all countries.

**Table 4:** Standardized maximum likelihood coefficients estimated for the hypothesized model by country.

Country	Sexism effect on myths	Myths effect on opposition	Total effect	Indirect effect	Direct effect
Austria	.49***	.61***	.46***	.24***	.22***
Australia	.51***	.74***	.36***	.26***	.10***
Bulgaria	.42***	.42***	.33***	.18***	.15***
Canada	.54***	.72***	.44***	.29***	.15***
Germany	.55***	.76***	.35***	.30***	.05*
Great Britain	.53***	.64***	.44***	.28***	.16***
Ireland	.53***	.66***	.46***	.28***	.18***
Israel	.42***	.49***	.41***	.18***	.22***
Japan	.28***	.36***	.25***	.07***	.18***
Norway	.65***	.81***	.49***	.42***	.06*
Poland	.50***	.50***	.56***	.25***	.31***
Russia	.35***	.46***	.30***	.12***	.17***
Slovenia	.51***	.60***	.44***	.26***	.17***
Spain	.45***	.37***	.57***	.20***	.36***
Sweden	.66***	.78***	.44***	.43***	.01, *ns*
USA	.55***	.59***	.46***	.31***	.15***

Significance of the indirect effects was estimated using bootstrap analyses with 1000 bootstrapping resamples.

* *p* < .02,

*** *p* < .001.

**Fig 1:**
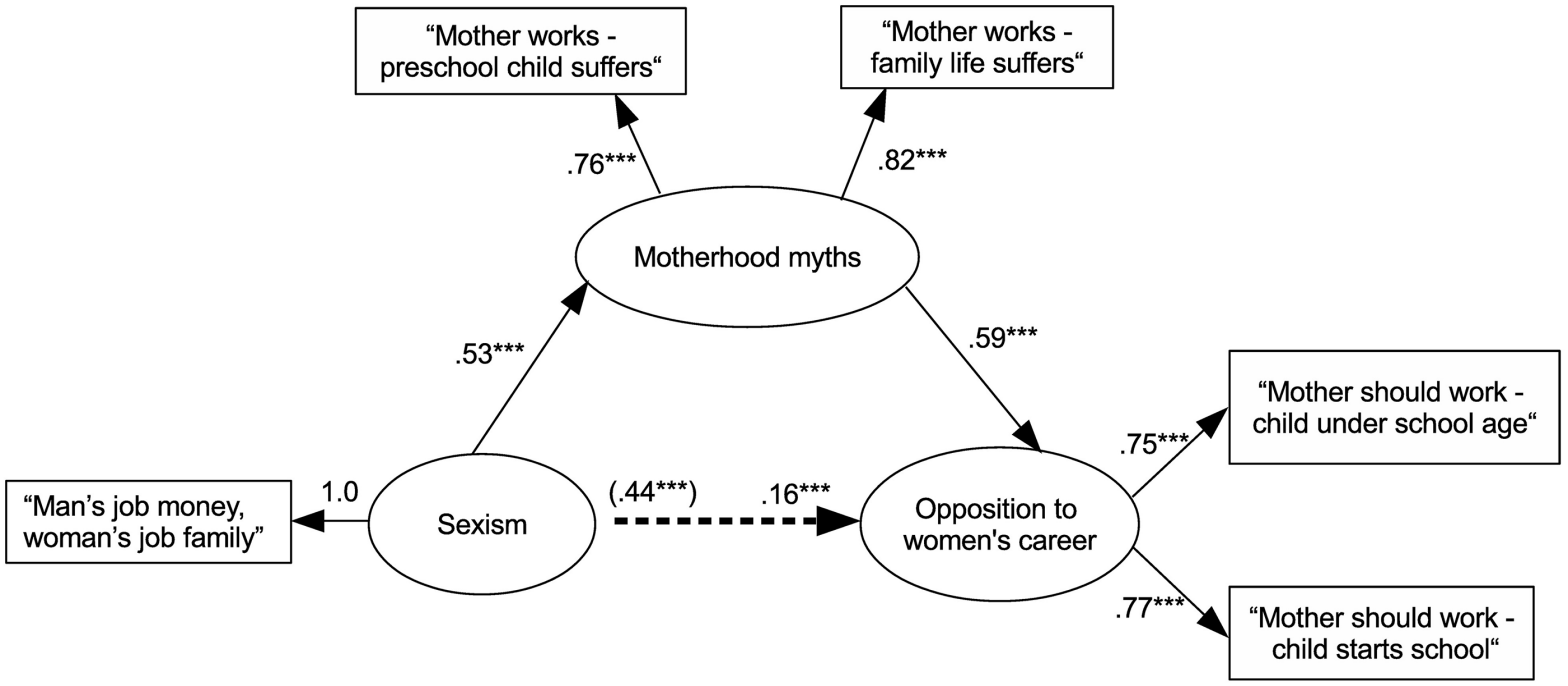
Standardized maximum likelihood coefficients for the structural equation model testing the relationship between sexism and opposition to women’s career, mediated by the endorsement of motherhood myths. The coefficient in parentheses represents parameter estimate for the total effect of prejudice on opposition to women’s career. *** *p* < .001.

In order to provide an overview of the proposed mediational model, we next present the analyses conducted on the total of the 16 countries retained. The hypothesized mediational model shows acceptable fit to the data, χ^2^(4, *N* = 38178) = 971.09, *p* < .001, CFI = .983, RMSEA = .08 [90% CI = .07, .08], SRMR = .04, AIC = 473476. Inspection of the fit indices of the first alternative model where endorsement of motherhood myths predicted sexism that, in turn, predicted opposition confirms that this alternative model shows poorer fit to the data than the proposed model, χ^2^(4, *N* = 38178) = 7583.1, *p* < .001, CFI = .870, RMSEA = .22 [90% CI = .21, .22], SRMR = .13, AIC = 480088. The second alternative model, where opposition to women’s career predicted motherhood myths shows poor fit to the data, χ^2^(5, *N* = 38178) = 14224.61, *p* < .001, CFI = .756, RMSEA = .27 [90% CI = .26, .27], SRMR = .21, AIC = 486728, and accordingly fits the data less well than the proposed mediational model, Δ χ^2^ (1, 38178) = 13254 *p* < .001. As can be seen in Fig 1, the standardized regression coefficient for the direct effect of sexism on opposition to women’s career is significant (*β* = .16, *p* < .001). In addition, the unstandardized estimate for the indirect effect excludes zero (.13, *SE* = 0.003, bias corrected 95% CI [.12, .13]) and, therefore, is significant. Taken together, analyses conducted on the whole sample, as well as on each country separately, support our main assumption that endorsement of motherhood myths is a significant mediator of the relationship between sexism and opposition to women’s career.

### Step 4. Moderated mediation analyses

#### Indirect effect through survey waves

The moderated mediation model was estimated using a multiple group approach. This model exhibits good fit to the data, χ^2^(6, *N* = 38178) = 438.88, *p* < .001, CFI = .992, RMSEA = .06 [90% CI = .05, .06], SRMR = .01. The standardized coefficients for the total effect are .50 in the 2012 survey, and .52 in the 1994 survey. The unstandardized estimates for the indirect effect is .10, *SE* = 0.003, bias corrected 95% CI [.10, .11] in the 2012 survey, and .11, *SE* = 0.003, bias corrected 95% CI [.10, .11] in the 1994 survey. The intervals do not include zero, indicating that motherhood myths are a significant mediator of the relationship between sexism and opposition to women’s career in both survey waves. The difference between the indirect effect in 2012 and 1994 is not significant (-.003, *SE* = 0.004, bias corrected 95% CI [.-.01, .00]). We repeated the moderated mediation analysis in each country. As can be seen in Table 5, the indirect effect reaches significance in each survey wave in all countries. The indirect effect is not moderated by the survey year, except in Great Britain where the indirect effect, although still significant, decreased between 1994 and 2012, and Bulgaria, Poland, and Russia where the indirect effect slightly increased between 1994 and 2012.

**Table 5:** Standardized maximum likelihood coefficients estimated for the total and indirect effects as a function of the survey wave.

Country	Total effect	Direct effect	Indirect effect	Δ Indirect effect
Austria				
2012 (986)	.66***	.44***	.21***	
1994 (877)	.45***	.15**	.29***	-.07, *ns*
Australia				
2012 (1225)	.50***	.14***	.36***	
1994 (1518)	.56***	.16***	.40***	-.04, *ns*
Bulgaria				
2012 (838)	.32***	.15**	.17***	
1994 (914)	.24***	.13**	.11***	.06, *p* = .*003*
Canada				
2012 (727)	.58***	.18***	.39***	
1994 (1098)	.59***	.21***	.37***	.02, *ns*
Germany				
2012 (1390)	.50***	.13***	.38***	
1994 (2882)	.54***	.15***	.39***	-.01, *ns*
Great Britain				
2012 (735)	.55***	.28***	.27***	
1994 (806)	.53***	.13**	.40***	-.13, *p* = .005
Ireland				
2012 (899)	.56***	.19***	.37***	
1994 (794)	.55***	.25***	.29***	.08, *ns*
Israel				
2012 (1043)	.52***	.32***	.20***	
1994 (1159)	.41***	.17***	.24***	-.04, *ns*
Japan				
2012 (826)	.35***	.22***	.13***	
1994 (1098)	.24***	.16***	.08***	.05, *ns*
Norway				
2012 (1190)	.58***	.15***	.43***	
1994 (1784)	.64***	.15***	.49***	-.06, *ns*
Poland				
2012 (970)	.69***	.19***	.34***	
1994 (1278)	.45***	.15***	.30***	.03, *p* = .029
Russia				
2012 (1303)	.30***	.15***	.15***	
1994 (1694)	.35***	.22***	.13***	.02, *p* = .023
Slovenia				
2012 (937)	.48***	.24***	.24***	
1994 (931)	.41***	.17***	.24***	.00, *ns*
Spain				
2012 (2189)	.58***	.35***	.23***	
1994 (2067)	.53***	.36***	.17***	.06, *ns*
Sweden				
2012 (862)	.56***	.09, *ns*	.46***	
1994 (1062)	.53***	.10*	.43***	.03, *ns*
USA				
2012 (915)	.52***	.21***	.30***	
1994 (1202)	.49***	.13***	.36***	-.05, *ns*

Significance of the indirect effects are estimated using bootstrap analyses with 1000 bootstrapping resamples.

* *p* < .05,

** *p* < .01,

*** *p* < .001.

#### Indirect effect as a function of the respondents’ gender

The moderated mediation model exhibits good fit to the data, χ^2^(6, *N* = 38124) = 402.46, *p* < .001, CFI = .993, RMSEA = .06 [90% CI = .05, .06], SRMR = .01. The total effect of sexism on opposition to women’s career is positive and significant for both men (*β* = .52, *p* < .001) and women (*β* = .50, *p* < .001). The standardized indirect effect of sexism on opposition to women’s career through motherhood myths is .27 in the male subsample, and .29 in the female subsample. The unstandardized estimates for the indirect effect is .11, *SE* = 0.003, bias corrected 95% CI [.10, 12] in the male sample, and .10, *SE* = 0.003, bias corrected 95% CI [.09, .10] in the female sample. The intervals do not include zero, indicating that motherhood myths are a significant mediator of the relationship between sexism and opposition to women’s career among both men and women respondents. The difference between the indirect effect among men and women is not statistically significant (.01, *SE* = 0.004, bias corrected 95% CI [.00, .01]). We repeated this analysis in each country separately (see Table 6). Results confirm that the indirect effect of sexism on opposition to women’s career through motherhood myths is not moderated by the respondents’ gender in 15 out of the 16 countries. The only exception is Poland. In this country, the indirect effect is stronger for the female than for the male respondents.

**Table 6:** Standardized maximum likelihood coefficients estimated for the total and indirect effects as a function of the respondents’ gender.

Country	Total effect	Direct effect	Indirect effect	Δ Indirect effect
Austria				
Female (1049)	.59***	.32***	.27***	
Male (814)	.52***	.24***	.27***	.00, *ns*
Australia				
Female (1370)	.51***	.15***	.38***	
Male (1342)	.54***	.16***	.36***	.02, *ns*
Bulgaria				
Female (1066)	.31***	.13**	.14***	
Male (685)	.29***	.16**	.15***	-.01, *ns*
Canada				
Female (960)	.61***	.23***	.37***	
Male (854)	.55***	.16***	.38***	-.01, *ns*
Germany				
Female (2130)	.54***	.12***	.41***	
Male (2142)	.52***	.12***	.39***	.02, *ns*
Great Britain				
Female (837)	.55***	.19***	.35***	
Male (704)	.53***	.26***	.27***	.08, *ns*
Ireland				
Female (995)	.49***	.16***	.33***	
Male (693)	.61***	.31**	.30***	.03, *ns*
Israel				
Female (1214)	.37***	.14***	.22***	
Male (985)	.53***	.34***	.18***	.04, *ns*
Japan				
Female (1068)	.27***	.15***	.11***	
Male (856)	.30***	.20***	.10***	.01, *ns*
Norway				
Female (1569)	.63***	.13***	.50***	
Male (1405)	.62***	.13***	.49***	.00, *ns*
Poland				
Female (1225)	.60***	.37***	.23***	
Male (1023)	.54***	.39***	.14***	.08, *p* = .004
Russia				
Female (1967)	.36***	.20***	.16***	
Male (1030)	.32***	.15***	.17***	.01, *ns*
Slovenia				
Female (1017)	.52***	.20***	.31***	
Male (850)	.48***	.16***	.32***	-.01, *ns*
Spain				
Female (2242)	.53***	.38***	.14***	
Male (2012)	.51***	.36***	.14***	.00, *ns*
Sweden				
Female (1020)	.56***	.06, *ns*	.50***	
Male (883)	.56***	.16***	.40***	.10, *ns*
USA				
Female (1192)	.48***	.13**	.31***	
Male (925)	.48***	.17***	.35***	-.03, *ns*

Significance of the indirect effects are estimated using bootstrap analyses with 1000 bootstrapping resamples.

** *p* < .005,

*** *p* < .001.

## Discussion

Using a large representative sample of respondents from various countries the present research documented a psychosocial process of justification of discrimination against working women with children. As a preliminary step, hierarchical regression analysis established that sexism and motherhood myths predict opposition to women’s work, over and above gender, partnership, education, social status, religiosity and political orientation. Furthermore, structural equation modellings on the whole sample, as well as on each country separately, confirmed our main hypothesis that endorsement of motherhood myths mediates the relationship between sexism and opposition to women’s career following a birth. In addition, test of the moderated mediation indicated that the indirect effect reaches significance in each survey wave in almost all countries examined without substantial difference. Only in Bulgaria, Poland, and Russia did the indirect effect slightly increase between 1994 and 2012, suggesting that motherhood myths is more a justification for the expression of sexism nowadays than in the late 20^th^ century. Great Britain shows a reverse pattern with a slight decrease of the indirect effect between the two waves. However, besides these minor variations, it should be noted that motherhood myths remain a significant mediator of the sexism-opposition to women’s career relationship in all countries. The present research also considered participants' gender as a potential moderator of the indirect effect, and results indicated that the process of justification of discrimination against working women does not differ as a function of the respondents' gender. The only exception to this finding is Poland where the indirect effect is indeed stronger among women than among men. An examination of the specific features of female employment in this country sheds some light on this result. Young women in Poland are better educated than young men and are more likely to have permanent employment than men [77]. At the same time however, working women spend on average two and a half hours per day on unpaid work more than men—which is reflected by the fact that more than 1 in 3 women reduce their paid hours to part-time, while only 1 in 10 men do the same—and are predominant users of parental leave [3]. It is noteworthy that reduced working hours (and long periods of leave) hinders female career progression through less training, fewer opportunities for advancement, occupational segregation, and lower wages [78, 79]. Accordingly, in Poland women earn 9% less than men (one of the lowest gender pay gap in OECD) but the pay gap reaches 22% by presence of children (above the OECD average of 16%; [77]). The fact that women appear even more inclined than men to rely on motherhood myths to justify gender discrimination is consistent with a system justification perspective [63]. Drawing on the logic of cognitive dissonance theory, system justification theory in its strong form posits that members of disadvantaged groups may be even more likely than members of advantaged groups to support existing social inequalities [64]. The rational is that members of disadvantaged groups would experience psychological discomfort stemming from the concurrent awareness of their ingroup's inferiority within the system, and of their ingroup's contribution to that system. Justification of the status quo would therefore reduce dissonance [80]. The finding that women strongly rely on motherhood myths to justify gender discrimination precisely in a country with strong motherhood penalty can be regarded as an expression of this system justification motive.

The present research sheds new light on the effect of macrolevel inequality on the justification of discrimination, and more broadly on the process of legitimation of gender inequalities [9, 81]. In a recent study, Yu and Lee [82] found a negative association between women’s relative status in society and support for gender equality at home. More specifically, the authors found that although respondents in countries with smaller gender gaps express greater support for women’s participation in the labour force, they still exhibit less approval for egalitarian gender roles within the household, in particular regarding the share of domestic chores and childcare. As an explanation, the authors argued that the less traditional the gender division of labour is in a society, the more people need to express their freedom of maintaining these roles and to defend the gender system, leading to the endorsement of gender differentiation in the private sphere. However, the present research allows an alternative explanation for this seemingly paradoxical finding to be suggested. At a macrolevel, higher gender equality conveys strong suppressive factors (which reduce the expression of prejudice) by demonstrating that the society promotes egalitarianism between women and men. In parallel, the gender specialization in the division of the household responsibilities and especially regarding childcare provides a strong justifying factor (which releases prejudice) by emphasising essential differences between gender groups [26]. Thus, the counterintuitive finding that the more egalitarian a society is, the less people support gender equality at home may indeed reflect an attempt to justify the release of genuine sexism. Conversely, it is likely that a less egalitarian society brings with it some degree of tolerance towards gender discrimination, reducing the need to rely on justifications to express sexism. A closer look at our results regarding Norway and Japan supports this view. Norway and Japan appears as especially contrasted regarding gender equality, in particular with regard to economic participation and opportunity [1]. According to the World Economic Forum, Norway has the second smallest gender gap in the world. In addition, gender equality promotion is frequently mobilised both in political debates and in mainstream society [55]. For its part, Japan ranks 101^st^ on the overall gender gap index, which makes Japan well below average compared to other advanced industrial countries [83]. Besides this gender gap, consistent research reports a unique trivialisation of anti-gender equality discourses in the media [84] and of gender-based discriminatory behaviours in the workplace, including sexual harassment [85]. Comparing the strength of the indirect effect of sexism on opposition to women’s career through motherhood myths in these two countries (Table 4), it is noteworthy that the coefficient is larger in Norway than in Japan. This result gives support to the assumption that macrolevel gender (in)equality affects the psychological process of justification at the individual level. Future studies should clarify how macrolevel inequalities impact societal norms, which in turn influence legitimation processes.

It is also worth noting that the justifying function of motherhood myths is established in all analysed countries despite some notable differences between parental leaves policies and practices. For instance, the United States are the only OECD country to offer no nationwide entitlement to paid leave, neither for mothers nor for fathers [86]. On the other hand, the Nordic nations, with Norway and Sweden in the lead, are in the vanguard of progressive policy-making regarding shared parental leave entitlement: Sweden was the first country in the world in 1974 to offer fathers the possibility of taking paid parental leave, quickly joined by Norway in 1978 [87]. More recently in 2007, Germany introduced a new law aiming at encouraging shared parental leave. In practice, the length of the financial support for parental leave can increase from 12 to 14 months provided that fathers use the parental benefit for at least 2 months. Recent research aiming at investigating whether German men who take parental leave are judged negatively in the workplace revealed that, in contrast with women who experience penalty for motherhood [40], fathers do not face backlash effect when they take a long parental leave [88]. The authors concluded that "gender role attitudes have changed". Tempering this view, the present study indicates that even in countries promoting incentives for fathers to take parental leave, motherhood myths—and specifically the belief that mother's work threatens the family—are still a justification for gender discrimination in the workplace. With regard to practices, it should be noted that shared parental leave policies, whose purpose is to foster gender equality in the labor market, often fail to meet this objective, with the majority of fathers actually taking the minimum length of leave entitlement, or no parental leave at all, and the majority of mothers still facing the majority of childcare [88]. Once again, more research is needed to document the process of mutual influences between changing family policies and the maintenance of the gender status quo via justifying beliefs.

### Limitations and future directions

Although the hypothesized mediational process is supported by the data, and is in line with previous experimental findings [19], conclusion regarding causality are necessarily limited due to the correlational nature of the research. We hope that these preliminary findings will open the way to experimental studies allowing for a conclusion on the direction of causality between variables and the further documenting of the behavioural consequences of the endorsement of motherhood myths. For instance, future studies should consider the extent to which motherhood myths interact with organizational norms to constrain the hiring and promotion of women. Castilla and Benard [89] showed that when an organization explicitly values meritocracy, managers favour a male employee over an equally qualified female employee. One explanation for this seemingly paradoxical results lies in the legitimation function of meritocracy [17] which is likely to release the expression of sexism. We suggest that when organizations promote egalitarian norms, or put differently, when organizations set suppression factors, then motherhood myths may serve as a justification for unequal gender treatment regarding career outcomes.

Due to constraints related to the availability of data in the ISSP base, only one indicator was used to capture sexism. This can be regarded as a limitation providing that sexism is typically defined as a complex construct [20]. We argue that measuring the gender differentiation component of sexism through a single item represents a valid approach, as suggested by previous research indicating that single-item measures may be as reliable as aggregate scales [90–94]. However, using a multiple-item measure of sexism in future studies would provide a more comprehensive examination of the relations between the different components of sexism and opposition to gender equality in the workplace.

The present research focused on opposition to mothers' work as an indicator of gender discrimination. However, evidence suggests that motherhood myths may justify discrimination towards women as a whole rather than mothers only. First, as previously mentioned social roles create gender expectations [95] so that all women are expected to become mothers [47]. Furthermore, research using implicit association test indicate that people automatically associate women with family role [96]. As a consequence, it is plausible that employers rely on motherhood myths to discriminate against women in general regarding recruitement, performance evaluation, and rewards, arguing that women will sooner or later be less involved in work and less flexible for advancement than men [97]. This justification is compatible with the employers' reluctance to hire women and promote them to the highest positions even in the absence of productivity differences [98].

### Practical implications

In this study we were able to document that motherhood myths are a widespread justification for gender discrimination in the workplace, including in countries with anti-discrimination laws and advanced family policies. From this regard, the present findings help understand the paradoxical effects of family-friendly policies on women's economic attainment. Mandel and Semyonov [99], using data from 20 countries, found evidence that family policies aimed at supporting women's economic independence, and including provision of childcare facilities and paid parental leaves, increase rather than decrease gender earning gaps. This unexpected effect is due to the fact that family policies are disproportionally used by mothers rather than fathers, with the consequence that mothers are concentrated in part-time employment, female-typed occupations, yet underrepresented in top positions. The authors concluded that "there are distinct limits to the scope for reducing gender wage inequality in the labor market as long as women bear the major responsibility for household duties and child care" (p. 965). We would add that there are strong barriers to the scope for attaining gender equality at home as long as motherhood myths are uncritically accepted and used as justification for unequal gender arrangements. Recent works provided evidence of the efficiency of interventions aimed at reducing sexist beliefs [100] and at recognizing everyday sexism [101]. In the same vain, interventions aimed at informing people that motherhood myths are socially constructed and maintained [33], and that they affect women's advancement and fathers' involvement [35], would represent a first step towards the reduction of discrimination by depriving individuals of a justification for gender inequalities.

The present research builds on and extends past findings by demonstrating that men and women rely on the belief that women’s work threatens the well-being of youth and family to justify discrimination against working women. If, at an individual level, this process allows discrimination to be exhibited without appearing prejudiced [10], at the group and societal levels, such a process may contribute to the legitimation and reinforcement of the hierarchical power structure [63]. By documenting a pervasive process by which people invoke motherhood myths to hinder women’s economic participation, the present research emphasizes the need to be vigilant about any attempts to promote a return to traditional gender roles, an issue of central importance given the contemporary rollback of women’s rights in advanced industrial countries [102].

## Supporting information

S1 Table Comparative test of the goodness of fit of the hypothesized measurement model vs. alternative measurement model. All differences are significant at *p* < .001.
(PDF)

S2 Table Test of the invariance of the measurement model across survey waves by country. In Poland and Slovenia partial metric invariance of the measurement model was attained by setting free the loading of the item “*Do you think that women should work outside the home full-time*, *part-time or not at all after the youngest child starts school*?” on the “opposition” latent variable. This partly constrained model show good fit indices in Poland, χ^2^(7, *N* = 2248) = 36.18, *p* = .006, CFI = .990, RMSEA = .06 [90% CI = .04, .08], and Slovenia, χ^2^(7, *N* = 1867) = 12.92, *p* = .058, *ns*, CFI = .999, RMSEA = .03 [90% CI = .00, .05]. In the USA, partial metric invariance of the measurement model was attained by setting free the loading of the item “*All in all*, *family life suffers when the woman has a full-time job*” on the “motherhood myths” latent variable, χ^2^(7, *N* = 2117) = 11.08, *p* = .069, *ns*, CFI = .999, RMSEA = .02 [90% CI = .00, .04].
(PDF)

S3 Table Test of the invariance of the measurement model across gender groups by count. (PDF)

S1 Supplementary Information Additional details concerning the way the research was conducted. (DOCX)
