# Correction: Justifying gender discrimination in the workplace: The mediating role of motherhood myths

**DOI:** 10.1371/journal.pone.0201150

**Published:** 2018-07-18

**Authors:** Catherine Verniers, Jorge Vala

There is an error in the first sentence of the “Goodness of fit of the models” section in the Results. The correct sentence is: Inspection of the fit indices indicates that the hypothesized model fits the data better than the first alternative model in 17 out of the 18 analyzed countries (Table 3).

There is an error in the third sentence of the “Goodness of fit of the models” section in the Results. The correct sentence is: The comparison of the fit indices indicates that the two models fit the data to almost the same extent in the remaining country (i.e., Philippines).

There are errors in the second paragraph of the “Test of the relationships between variables” section in the Results. The correct paragraph is: In order to provide an overview of the proposed mediational model, we next present the analyses conducted on the total of the 17 countries retained. The hypothesized mediational model shows acceptable fit to the data, χ^2^(3, *N* = 40708) = 358.62, *p* < .001, CFI = .994, RMSEA = .05 [90% CI = .04, .05], SRMR = .01, AIC = 507004. Inspection of the fit indices of the first alternative model where endorsement of motherhood myths predicted sexism that, in turn, predicted opposition confirms that this alternative model shows poorer fit to the data than the proposed model, χ^2^(4, *N* = 40708) = 5043.38, *p* < .001, CFI = .917, RMSEA = .17 [90% CI = .17, .18], SRMR = .10, AIC = 511687, Δ χ^2^ (1, 40708) = 4684.8 *p* < .001. The second alternative model, where opposition to women’s career predicted motherhood myths shows poor fit to the data, χ^2^(5, *N* = 40708) = 14000.04, *p* < .001, CFI = .769, RMSEA = .26 [90% CI = .25, .26], SRMR = .21, AIC = 520641, and accordingly fits the data less well than the proposed mediational model, Δ χ^2^ (1, 40708) = 13641 *p* < .001. As can be seen in Fig 1, the standardized regression coefficient for the direct effect of sexism on opposition to women’s career is significant (*β* = .23, *p* < .001). In addition, the unstandardized estimate for the indirect effect excludes zero (.11, SE = 0.002, bias corrected 95% CI [.10, .11]) and, therefore, is significant. Taken together, analyses conducted on the whole sample, as well as on each country separately, support our main assumption that endorsement of motherhood myths is a significant mediator of the relationship between sexism and opposition to women’s career.

Fig 1 is incorrect and the caption for Fig 1 is incomplete. The authors have provided a corrected Fig 1 and a complete, correct Fig 1 caption here.

**Fig 1:**
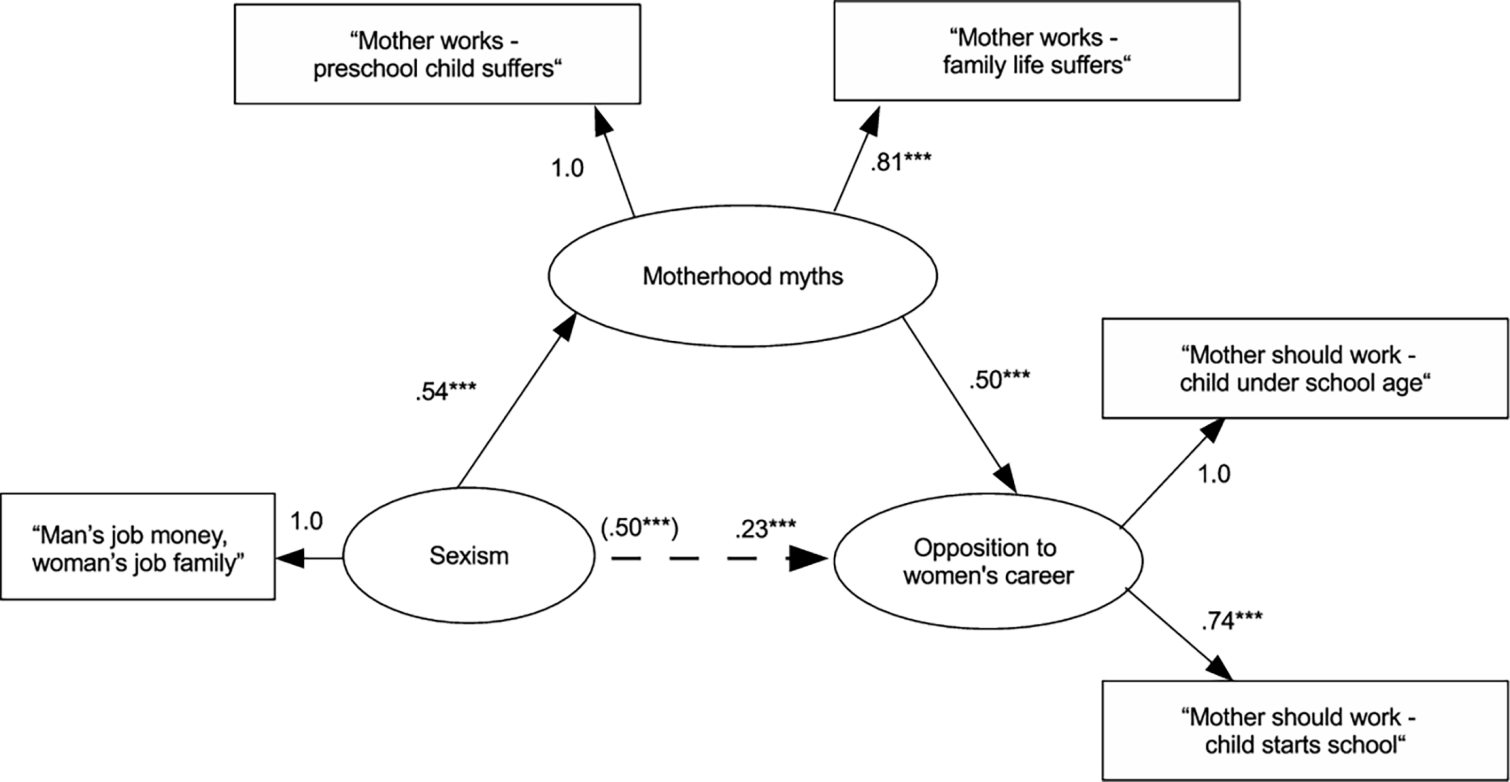
Standardized maximum likelihood coefficients for the structural equation model testing the relationship between sexism and opposition to women’s career, mediated by the endorsement of motherhood myths. The loading of the single indicator of the sexism variable and the loading of the first indicator of the motherhood myths and opposition variables are constrained to 1.00. The coefficient in parentheses represents parameter estimate for the total effect of prejudice on opposition to women’s career. *** *p* < .001.

Table 3 is incorrect. The authors have provided a corrected version here.

**Table 3:** Goodness-of-fit indices for the hypothesized mediational model and alternative models by country.

Country	χ ^2^	CFI	RMSEA	SRMR	AIC	Δ χ ^2^
Austria						
Hypothesized model (df = 3)	48.42	.979	.09 [.06, .11]	.02	22973	
Alternative model 1 (df = 4)	216.76	.900	.16 [.15, .18]	.09	23139	168.3
Alternative model 2 (df = 5)	658.16	.694	.26 [.24, .28]	.20	23578	609.7
Australia						
Hypothesized model (df = 3)	48.11	.991	.07 [.05, .09]	.01	30100	
Alternative model 1 (df = 4)	674.91	.860	.24 [.23, .26]	.14	30725	626.8
Alternative model 2 (df = 5)	1024.1	.787	.27 [.25, .28]	.22	31072	975.9
Bulgaria						
Hypothesized model (df = 3)	16.98	.989	.05 [.03, .07]	.01	24157	
Alternative model 1 (df = 4)	155.14	.885	.14 [.12, .16]	.09	24293	138.1
Alternative model 2 (df = 5)	290.97	.782	.18 [.16, .19]	.13	24427	273.99
Canada						
Hypothesized model (df = 3)	37.11	.990	.07 [.05, .10]	.01	21349	
Alternative model 1 (df = 4)	481.23	.862	.25 [.23, .27]	.14	21791	444.1
Alternative model 2 (df = 5)	736.87	.789	.28 [.26, .30]	.23	22045	699.7
Czech Republic						
Hypothesized model (df = 3)	12.85	.997	.03 [.01, .05]	.01	32739	
Alternative model 1 (df = 4)	223.65	.924	.14 [.13, .16]	.10	32948	210.7
Alternative model 2 (df = 5)	370.17	.874	.17 [.15, .18]	.13	33092	357.3
Germany						
Hypothesized model (df = 3)	124.93	.985	.09 [.08, .11]	.01	51390	
Alternative model 1 (df = 4)	1117.9	.862	.25 [.24, .26]	.14	52381	992.9
Alternative model 2 (df = 5)	1771.5	.781	.28 [.27, .29]	.23	53033	1646.6
Great Britain						
Hypothesized model (df = 3)	52.72	.980	.10 [.08, .12]	.02	16887	
Alternative model 1 (df = 4)	275.4	.892	.21 [.18, .23]	.12	17108	222.6
Alternative model 2 (df = 5)	616.97	.757	.28 [.26, .30]	.22	17447	564.2
Ireland						
Hypothesized model (df = 3)	22.24	.994	.06 [.03, .08]	.01	20263	
Alternative model 1 (df = 4)	322.73	.898	.21 [.19, .23]	.12	20561	300.4
Alternative model 2 (df = 5)	712.91	.772	.28 [.27, .30]	.23	20949	690.6
Israel						
Hypothesized model (df = 3)	17.96	.994	.04 [.02, .07]	.01	26052	
Alternative model 1 (df = 4)	226.37	.906	.15 [.14, .17]	.10	26258	208.4
Alternative model 2 (df = 5)	505.04	.788	.21 [.19, .22]	.16	26535	487
Japan						
Hypothesized model (df = 3)	26.12	.984	.06 [.04, .08]	.02	26339	
Alternative model 1 (df = 4)	113.88	.926	.11 [.10, .13]	.08	26424	87.75
Alternative model 2 (df = 5)	214.43	.859	.14 [.13, .16]	.10	26523	188.3
Norway						
Hypothesized model (df = 3)	50.45	.993	.07 [.05, .09]	.01	32416	
Alternative model 1 (df = 4)	891.07	.865	.27 [.25, .28]	.14	33254	840.6
Alternative model 2 (df = 5)	1558.4	.764	.32 [.31, .33]	.27	33920	1508
Philippines						
Hypothesized model (df = 3)	16.45	.986	.04 [.02, .06]	.01	29706	
Alternative model 1 (df = 4)	31.9	.970	.05 [.03, .07]	.03	29719	15.4
Alternative model 2 (df = 5)	180.81	.814	.12 [.10, .14]	.08	29866	164.3
Poland						
Hypothesized model (df = 3)	30.66	.991	.06 [.04, .08]	.01	28411	
Alternative model 1 (df = 4)	166.52	.948	.13 [.11, .15]	.07	28545	135.8
Alternative model 2 (df = 5)	993.28	.683	.29 [.28, .31]	.22	29369	962.6
Russia						
Hypothesized model (df = 3)	11.86	.997	.03 [.01, .05]	.00	35329	
Alternative model 1 (df = 4)	325.42	.882	.16 [.14, .17]	.11	35640	313.5
Alternative model 2 (df = 5)	387.38	.859	.16 [.14, .17]	.12	35700	375.5
Slovenia						
Hypothesized model (df = 3)	4.83	.999	.01 [.00, .04]	.00	22546	
Alternative model 1 (df = 4)	362.28	.889	.21 [.20, .23]	.13	22902	357.4
Alternative model 2 (df = 5)	595.08	.817	.25 [.23, .26]	.21	23133	590.2
Spain						
Hypothesized model (df = 3)	47.87	.992	.05 [.04, .07]	.01	48461	
Alternative model 1 (df = 4)	303.57	.945	.13 [.12, .14]	.08	48715	255.7
Alternative model 2 (df = 5)	1388.5	.746	.25 [.24, .26]	.19	49798	1340.6
Sweden						
Hypothesized model (df = 3)	41.46	.990	.08 [.06, .10]	.01	20646	
Alternative model 1 (df = 4)	425.37	.887	.23 [.21, .25]	.12	21027	383.9
Alternative model 2 (df = 5)	994.45	.735	.32 [.30, .33]	.25	21595	952.9
USA						
Hypothesized model (df = 3)	2.70	1.00	.00 [.00, .03]	.00	25313	
Alternative model 1 (df = 4)	354.48	.889	.20 [.18, .22]	.12	25663	351.7
Alternative model 2 (df = 5)	683.44	.785	.25 [.23, .26]	.20	25990	680.7

Δ χ^2^ compares each alternative model with the hypothesized mediational model. All Δ χ^2^ tests are significant at *p* < .001.

Table 4 is incorrect. The authors have provided an updated Table 4 here.

**Table 4:** Standardized maximum likelihood coefficients estimated for the hypothesized model by country.

Country	Sexism effect on myths	Myths effect on opposition	Total effect	Indirect effect	Direct effect
Austria	.53***	.50***	.57***	.27***	.30***
Australia	.56***	.68***	.54***	.38***	.15***
Bulgaria	.41***	.44***	.33***	.18***	.14***
Canada	.57***	.67***	.58***	.38***	.19***
Czech Republic	.31***	.38***	.37***	.12***	.25***
Germany	.60***	.69***	.53***	.41***	.12***
Great Britain	.57***	.56***	.55***	.32***	.22***
Ireland	.57***	.57***	.58***	.33***	.24***
Israel	.44***	.46***	.45***	.20***	.24***
Japan	.30***	.33***	.29***	.10***	.18***
Norway	.66***	.75***	.62***	.50***	.12***
Poland	.56***	.37***	.61***	.20***	.40***
Russia	.34***	.47***	.33***	.16***	.17***
Slovenia	.52***	.58***	.50***	.30***	.19***
Spain	.46***	.35***	.54***	.16***	.37***
Sweden	.67***	.69***	.56***	.46***	.10**
USA	.55***	.59***	.48***	.33***	.14***

Significance of the indirect effects was estimated using bootstrap analyses with 1000 bootstrapping resamples.

** *p* < .005

*** *p* < .001.
