# Supplementary material for: Justifying gender discrimination in the workplace: The mediating role of motherhood myths
Source: PLoS One. 2018 Jan 9;13(1):e0190657. doi: 10.1371/journal.pone.0190657 (PMC5760038; doi:10.1371/journal.pone.0190657)
Supplement: S1 Table — All differences are significant at p < .001. (PDF) [file pone.0190657.s001.pdf]

S1 Table. Comparative test of the goodness of fit of the hypothesized measurement model vs. alternative measurement model.

Country	Model comparison (one latent variable model – three latent variables model)
Austria	$\Delta\chi^2$ (2, 1863) = 161.48
Australia	$\Delta\chi^2$ (2, 2743) = 222.1
Bulgaria	$\Delta\chi^2$ (2, 1752) = 210.41
Canada	$\Delta\chi^2$ (2, 1825) = 186.43
Czech Republic	$\Delta\chi^2$ (2, 2530) = 606.03
Germany	$\Delta\chi^2$ (2, 4272) = 492.6
Great Britain	$\Delta\chi^2$ (2, 1541) = 219.17
Ireland	$\Delta\chi^2$ (2, 1693) = 273.37
Israel	$\Delta\chi^2$ (2, 2202) = 353.58
Japan	$\Delta\chi^2$ (2, 1924) = 423.42
Norway	$\Delta\chi^2$ (2, 2974) = 252.16
Philippines	$\Delta\chi^2$ (2, 2289) = 307.28
Poland	$\Delta\chi^2$ (2, 2248) = 353.46
Russia	$\Delta\chi^2$ (2, 2997) = 437.97
Slovenia	$\Delta\chi^2$ (2, 1867) = 341.03
Spain	$\Delta\chi^2$ (2, 4256) = 902.06
Sweden	$\Delta\chi^2$ (2, 1904) = 248.33
USA	$\Delta\chi^2$ (2, 2117) = 319.38

All differences are significant at $p < .001$.
