# Supplementary material for: Justifying gender discrimination in the workplace: The mediating role of motherhood myths
Source: PLoS One. 2018 Jan 9;13(1):e0190657. doi: 10.1371/journal.pone.0190657 (PMC5760038; doi:10.1371/journal.pone.0190657)
Supplement: S2 Table — In Poland and Slovenia partial metric invariance of the measurement model was attained by setting free the loading of the item “Do you think that women should work outside the home full-time, part-time or not at all after the youngest child starts school?” on the “opposition” latent variable. This partly constrained model show good fit indices in Poland, χ2(7, N = 2248) = 36.18, p = .006, CFI = .990, RMSEA = .06 [90% CI = .04, .08], and Slovenia, χ2(7, N = 1867) = 12.92, p = .058, ns, CFI = .999, RMSEA = .03 [90% CI = .00, .05]. In the USA, partial metric invariance of the measurement model was attained by setting free the loading of the item “All in all, family life suffers when the woman has a full-time job” on the “motherhood myths” latent variable, χ2(7, N = 2117) = 11.08, p = .069, ns, CFI = .999, RMSEA = .02 [90% CI = .00, .04]. (PDF) [file pone.0190657.s002.pdf]

S2 Table. Test of the invariance of the measurement model across survey waves by country.

	Measurement model		
Country	Configural invariance	Metric invariance	
Austria	$\chi^2(6, 1863) = 59.66$<br>CFI = .974, RMSEA = .09	$\chi^2(8, 1863) = 66.96$<br>CFI = .972, RMSEA = .08	Δ CFI = .002
Australia	$\chi^2(6, 2743) = 70.84$<br>CFI = .986, RMSEA = .08	$\chi^2(8, 2743) = 71.23$<br>CFI = .986, RMSEA = .07	Δ CFI = .000
Bulgaria	$\chi^2(6, 1752) = 34.66$<br>CFI = .975, RMSEA = .07	$\chi^2(8, 1752) = 41.88$<br>CFI = .971, RMSEA = .07	Δ CFI = .004
Canada	$\chi^2(6, 1825) = 38.66$<br>CFI = .991, RMSEA = .07	$\chi^2(8, 1825) = 39.19$<br>CFI = .991, RMSEA = .06	Δ CFI = .000
Czech Republic	$\chi^2(6, 2530) = 13.04$<br>CFI = .997, RMSEA = .03	$\chi^2(8, 2530) = 26.77$<br>CFI = .993, RMSEA = .04	Δ CFI = .004
Germany	$\chi^2(6, 4272) = 126.28$<br>CFI = .984, RMSEA = .09	$\chi^2(8, 4272) = 129.87$<br>CFI = .983, RMSEA = .08	Δ CFI = .001
Great Britain	$\chi^2(6, 1541) = 51.15$<br>CFI = .982, RMSEA = .09	$\chi^2(8, 1541) = 53.66$<br>CFI = .982, RMSEA = .08	Δ CFI = .000
Ireland	$\chi^2(6, 1693) = 38.91$<br>CFI = .989, RMSEA = .08	$\chi^2(8, 1693) = 55.63$<br>CFI = .984, RMSEA = .08	Δ CFI = .005
Israel	$\chi^2(6, 2202) = 22.4$<br>CFI = .993, RMSEA = .05	$\chi^2(8, 2202) = 23.63$<br>CFI = .994, RMSEA = .04	Δ CFI = -.001
Japan	$\chi^2(6, 1924) = 27.91$<br>CFI = .986, RMSEA = .06	$\chi^2(8, 1924) = 28.10$<br>CFI = .987, RMSEA = .05	Δ CFI = -.001

Norway	χ^2(6, 2974) = 51.03<br>CFI = .992, RMSEA = .07	χ^2(8, 2974) = 67.42<br>CFI = .990, RMSEA = .07	Δ CFI = .002
Philippines	χ^2(6, 2289) = 6.90<br>CFI = 999, RMSEA = .01	χ^2(8, 2289) = 11.18<br>CFI = .997, RMSEA = .01	Δ CFI = .002
Poland	χ^2(6, 2248) = 28.71<br>CFI = .992, RMSEA = .05	χ^2(8, 2248) = 93.51<br>CFI = .970, RMSEA = .09	Δ CFI = .022
Russia	χ^2(6, 2997) = 22.69<br>CFI = .993, RMSEA = .04	χ^2(8, 2997) = 27.94<br>CFI = .991, RMSEA = .04	Δ CFI = .002
Slovenia	χ^2(6, 1867) = 9.33<br>CFI = .999, RMSEA = .02	χ^2(8, 1867) = 75.17<br>CFI = .974, RMSEA = .09	Δ CFI = .025
Spain	χ^2(6, 4256) = 39.24<br>CFI = .994, RMSEA = .54	χ^2(8, 4256) = 71.12<br>CFI = .989, RMSEA = .06	Δ CFI = .005
Sweden	χ^2(6, 1904) = 43.63<br>CFI = .989, RMSEA = .08	χ^2(8, 1904) = 44.40<br>CFI = .989, RMSEA = .06	Δ CFI = .000
USA	χ^2(6, 2117) = 7.78<br>CFI = .999, RMSEA = .01	χ^2(8, 2117) = 60.43<br>CFI = .984, RMSEA = .07	Δ CFI = .015

In Poland and Slovenia partial metric invariance of the measurement model was attained by setting free the loading of the item "*Do you think that women should work outside the home full-time, part-time or not at all after the youngest child starts school?*" on the "opposition" latent variable. This partly constrained model show good fit indices in Poland, $\chi^2(7, N = 2248) = 36.18$, $p = .006$, CFI = .990, RMSEA = .06 [90% CI = .04, .08], and Slovenia, $\chi^2(7, N = 1867) = 12.92$, $p = .058$, *ns*, CFI = .999, RMSEA = .03 [90% CI = .00, .05]. In the USA, partial metric invariance of the measurement model was attained by setting free the loading of the item "*All in all, family life suffers when the woman has a full-time job*" on the "motherhood myths" latent variable, $\chi^2(7, N = 2117) = 11.08$, $p = .069$, *ns*, CFI = .999, RMSEA = .02 [90% CI = .00, .04].
