# Supplementary material for: Justifying gender discrimination in the workplace: The mediating role of motherhood myths
Source: PLoS One. 2018 Jan 9;13(1):e0190657. doi: 10.1371/journal.pone.0190657 (PMC5760038; doi:10.1371/journal.pone.0190657)
Supplement: S3 Table — (PDF) [file pone.0190657.s003.pdf]

S3 Table. Test of the invariance of the measurement model across gender groups by country.

	Measurement model		
Country	Configural invariance	Metric invariance	
Austria	χ^2(6, 1863) = 54.09<br>CFI = .977, RMSEA = .09	χ^2(8, 1863) = 63.53<br>CFI = .974, RMSEA = .08	Δ CFI = .003
Australia	χ^2(6, 2712) = 50.54<br>CFI = .990, RMSEA = .07	χ^2(8, 2712) = 51.70<br>CFI = .991, RMSEA = .06	Δ CFI = -.001
Bulgaria	χ^2(6, 1751) = 23.99<br>CFI = .986, RMSEA = .05	χ^2(8, 1751) = 24.32<br>CFI = .988, RMSEA = .04	Δ CFI = -.002
Canada	χ^2(6, 1814) = 45.53<br>CFI = .988, RMSEA = .08	χ^2(8, 1814) = 47.38<br>CFI = .988, RMSEA = .07	Δ CFI = .000
Czech Republic	χ^2(6, 2530) = 15.32<br>CFI = .997, RMSEA = .03	χ^2(8, 2530) = 19.7<br>CFI = .996, RMSEA = .03	Δ CFI = .001
Germany	χ^2(6, 4272) = 138.14<br>CFI = .983, RMSEA = .10	χ^2(8, 4272) = 145.44<br>CFI = .983, RMSEA = .09	Δ CFI = .000
Great Britain	χ^2(6, 1541) = 56.26<br>CFI = .980, RMSEA = .10	χ^2(8, 1541) = 57.33<br>CFI = .980, RMSEA = .08	Δ CFI = .000
Ireland	χ^2(6, 1688) = 24.53<br>CFI = .994, RMSEA = .06	χ^2(8, 1688) = 27.56<br>CFI = .994, RMSEA = .05	Δ CFI = .000
Israel	χ^2(6, 2199) = 21.58<br>CFI = .993, RMSEA = .04	χ^2(8, 2199) = 22.95<br>CFI = .994, RMSEA = .04	Δ CFI = -.001
Japan	χ^2(6, 1924) = 26.89<br>CFI = .986, RMSEA = .06	χ^2(8, 1924) = 30.58<br>CFI = .985, RMSEA = .05	Δ CFI = .001

Norway	$\chi^2(6, 2974) = 64.25$<br>CFI = .991, RMSEA = .08	$\chi^2(8, 2974) = 66.3$<br>CFI = .991, RMSEA = .07	Δ CFI = .000
Philippines	$\chi^2(6, 2289) = 27.48$<br>CFI = .977, RMSEA = .05	$\chi^2(8, 2289) = 30.08$<br>CFI = .977, RMSEA = .05	Δ CFI = .000
Poland	$\chi^2(6, 2248) = 30.88$<br>CFI = .992, RMSEA = .06	$\chi^2(8, 2248) = 38.45$<br>CFI = .990, RMSEA = .05	Δ CFI = .002
Russia	$\chi^2(6, 2997) = 16.14$<br>CFI = .996, RMSEA = .03	$\chi^2(8, 2997) = 16.9$<br>CFI = .997, RMSEA = .02	Δ CFI = -.001
Slovenia	$\chi^2(6, 1867) = 10.39$<br>CFI = .999, RMSEA = .02	$\chi^2(8, 1867) = 11.48$<br>CFI = .999, RMSEA = .02	Δ CFI = .000
Spain	$\chi^2(6, 4254) = 48.7$<br>CFI = .992, RMSEA = .05	$\chi^2(8, 4254) = 54.37$<br>CFI = .991, RMSEA = .05	Δ CFI = .001
Sweden	$\chi^2(6, 1903) = 51.81$<br>CFI = .988, RMSEA = .09	$\chi^2(8, 1903) = 53.35$<br>CFI = .988, RMSEA = .07	Δ CFI = .000
USA	$\chi^2(6, 2117) = 15.50$<br>CFI = .997, RMSEA = .04	$\chi^2(8, 2117) = 27.43$<br>CFI = .994, RMSEA = .05	Δ CFI = .003
