# Supplementary material for: Justifying gender discrimination in the workplace: The mediating role of motherhood myths
Source: PLoS One. 2018 Jan 9;13(1):e0190657. doi: 10.1371/journal.pone.0190657 (PMC5760038; doi:10.1371/journal.pone.0190657)
Supplement: S1 Supplementary Information — (DOCX) [file pone.0190657.s004.docx]

**S1 Supporting information. Additional details concerning the way the research was conducted.**

Regarding data selection, we had strong reasons to rely on the International Social Survey Programme. First, the ISSP Family and changing gender roles module provides highly relevant data for our research question, allowing both cross-national and cross-time comparisons. In addition, all anonymized data and documentation are freely and publicly made available.

The 2012 survey was the fourth of the Family and changing gender roles module. Previous surveys have been conducted in 1988, 1994, and 2002. The decision to retain the 1994 and 2012 waves was made prior to analyses. The decision was based on a compromise between the number of countries available for comparison (5 for the 1988 wave, 18 for the 1994 wave, and 26 for the 2002 wave), and the elapsed time between the waves (nearly one generation between the 1994 and the 2012 waves, 10 years between the 2002 and the 2012 waves). Considering our research question regarding the potential effect of time on the hypothesized mediational model, and our interest in taking into account various countries with contrasted policies regarding gender-discrimination, we chose to retain the 1994 (the second wave) as the baseline and the 2012 (the fourth/last wave) for comparison. We downloaded the 2012 and 1994 data files from the Gesis data archive website.

Although one aim of the ISSP is to secure continuity across survey waves, the two databases differ in two main respects: Country participation, and the number of items included. To allow comparisons between countries in the two waves, we selected the 18 countries that participated in both survey waves. Accordingly the following countries which did not participate in both waves were not retained for analysis: Argentina, Chile, China, Croatia, Denmark, Finland, France, Hungary, Iceland, India, Italy, Latvia, Lithuania, Netherlands, New Zealand, Northern Ireland, Slovakia, South Africa, South Korea, Switzerland, Taiwan, Turkey, Venezuela. No other observations were eliminated.

As for the selection of variables, the ISSP 2012 questionnaire contains 63 variables divided into 12 topics (i.e., attitudes towards family and gender roles; attitudes towards marriage; alternative family forms; attitudes towards children; gender, care and social policy; preferred and actual division of paid and unpaid work: model families; income in partnership; gendered division of household work; power and decision making within partnership; work-family conflict; happiness and satisfaction; additional demographics/ background variables). Amongst the 63 variables, 27 are common to the 2012 and 1994 waves. These variables have been thoroughly considered to determine which ones were theoretically-sound indicators for the concepts under examination (i.e., gender differentiation, threat posed to the family by mother's work, opposition to women's career). The theory-driven process led to the selection of five variables. It should be noted that the items pertaining to these five variables were presented in the first quarter of the questionnaire in both waves, which we think limits to some extent the effect of the variability in the questionnaires' length and content (see above).
